# Retraction Note: Psoriasin (S100A7) is a novel biomarker for lung squamous cell carcinoma in humans

**DOI:** 10.1186/s12935-016-0316-3

**Published:** 2016-05-31

**Authors:** Guijuan Liu, Qiang Wu, Guilan Liu, Xueying Song, Jihong Zhang

**Affiliations:** Department of Cytology Laboratory, People’s Hospital of Linyi, Linyi, 276300 Shandong China; Department of Nursing, People’s Hospital of Yinan, Linyi, 276300 Shandong China; Department of Nursing, People’s Hospital of Yinan, Linyi, 276300 Shandong China; Department of Thoracic Surgery, the People’s Hospital of Rizhao, Rizhao, China

## Retraction to: Cancer Cell International (2015) 15:18 DOI 10.1186/s12935-014-0154-0

The editors are retracting this article [1] as it has been previously published in the *International Journal of Clinical and Experimental Pathology* [2] and it is therefore a redundant publication. The authors could not be reached by the journal for comment on the retraction.

## References

[1] Liu G, Wu Q, Liu G, Song X, Zhang J (2015). Psoriasin (S100A7) is a novel biomarker for lung squamous cell carcinoma in humans. Cancer Cell Int.

[2] Liu G, Wu Q, Liu G, Song X, Zhang J (2014). Knockdown of S100A7 reduces lung squamous cell carcinoma cell growth in vitro and in vivo. Int J Clin Exp Pathol.

